# Heparanase Inhibition Reduces Glucose Levels, Blood Pressure, and Oxidative Stress in Apolipoprotein E Knockout Mice

**DOI:** 10.1155/2017/7357495

**Published:** 2017-10-26

**Authors:** Shadi Hamoud, Rabia Shekh Muhammad, Niroz Abu-Saleh, Ahmad Hassan, Yaniv Zohar, Tony Hayek

**Affiliations:** ^1^Internal Medicine E Department, Rambam Health Care Campus and Rappaport Faculty of Medicine, Technion, Haifa, Israel; ^2^Internal Medicine A Department, Rambam Health Care Campus and Rappaport Faculty of Medicine, Technion, Haifa, Israel; ^3^Lipid Research Laboratory, Rappaport Faculty of Medicine, Technion, Haifa, Israel; ^4^Pathology Department, Rambam Health Care Campus and Rappaport Faculty of Medicine, Technion, Haifa, Israel

## Abstract

**Background:**

Atherosclerosis is a multifactorial process. Emerging evidence highlights a role of the enzyme heparanase in various disease states, including atherosclerosis formation and progression.

**Objective:**

The aim of the study was to investigate the effect of heparanase inhibition on blood pressure, blood glucose levels, and oxidative stress in apoE−/− mice.

**Methods:**

Male apoE−/− mice were divided into two groups: one treated by the heparanase inhibitor PG545, administered intraperitoneally weekly for seven weeks, and the other serving as control group (injected with saline). Blood pressure was measured a day before sacrificing the animals. Serum glucose levels and lipid profile were measured. Assessment of oxidative stress was performed as well.

**Results:**

PG545 significantly lowered blood pressure and serum glucose levels in treated mice. It also caused significant reduction of the serum oxidative stress. For safety concerns, liver enzymes were assessed, and PG545 caused significant elevation only of alanine aminotransferase, but not of the other hepatic enzymes.

**Conclusion:**

Heparanase inhibition by PG545 caused marked reduction of blood pressure, serum glucose levels, and oxidative stress in apolipoprotein E deficient mice, possibly via direct favorable metabolic and hemodynamic changes caused by the inhibitor. Possible hepatotoxic and weight wasting effects are subject for future investigation.

## 1. Background

Atherosclerosis is a multifactorial process and is accelerated in the presence of several medical conditions-risk factors, such as diabetes mellitus, hypertension, dyslipidemia, and tobacco abuse.

Oxidative stress (OS) is involved in the pathogenesis and progression of atherosclerosis and vascular endothelial disorders, by promoting lipid peroxidation of serum lipoproteins, then enhancing their proatherogenic properties [1, 2]. Oxidative stress is known to be elevated in several diseases, such as diabetes mellitus, hypertension, and hyperlipidemia, and is usually associated with poor control and more frequent disease-related complications. Several antioxidants have been proven to protect against OS and the resulting attenuation of the atherosclerotic process progression. These antioxidants could be endogenous, such as the enzymes superoxide dismutase, catalase, and glutathione reductase, and exogenous, such as the nutritional supplements tocopherols, ascorbic acid, carotenoids and polyphenols [3, 4], extracts from red wine [5, 6], pomegranate juice [7–10], and licorice roots [11, 12], as well as niacin (nicotinic acid) [13].

Heparan sulphate proteoglycans (HSPGs) are cell-surface and extracellular matrix (ECM) macromolecules that are composed of glycosaminoglycan chains covalently linked to a protein core. Different modifications in the protein skeleton and in the disaccharide chains are responsible for the wide variety of the HSPGs, which are characterized from each other by their function and their biochemical properties. Heparan sulphate has the ability to bind variable matrix proteins, collagen, laminin, and fibronectin, and plays an important role in cellular-extracellular matrix interactions [14–16]. As an important protein binder, heparan sulphate has important roles both in normal biologic processes, like cell differentiation [17, 18], tissue repair [19, 20], cell adhesion [21], cell migration, and tissue forming in the fetus [22], and in pathologic processes, like tumor formation and metastatic spread [17, 18, 23–25], angiogenesis in malignant tumors [26, 27], autoimmune processes and inflammatory reactions [28–30], diabetes [31], kidney dysfunction [32, 33], and vessel wall pathologies: thrombosis and atherosclerosis [34–36]. The bidirectional interaction between HSPGs and the ECM is important for keeping the continuity and integrity of tissues and organs. Degradation of the HSPGs may affect the integrity and function of tissues and causes a mechanism for cells to interact with changes in the ECM. Enzymatic breakdown of heparan sulphate may be a part of multiple basic processes, like pregnancy and morphogenesis, inflammation, angiogenesis, and spread of malignant tumors. Heparanase, an endo-*β*-glucosidase, is the enzyme that cleaves heparan sulphate chains in specific sites along the polysaccharide chain [37–40]. In mammals, a dominant heparanase exists, as was proved in biochemical investigations and colonization of the gene encoding heparanase [18, 24]. Multiple human cell lines were shown to have heparanase activity, including trophoblast cells, platelets, mast cells, neutrophils, macrophages, and T and B cell lymphocytes, as well as cells of different malignant tumors, like lymphoma, melanoma, and carcinoma [18, 19, 27]. The active form of heparanase is a heterodimer, composed of two subunits, 50 and 8 kDa molecular weight [41]. In their review, Vlodavsky et al. [23] reported the involvement of heparanase in vessel wall disorders. Baker et al. [42] reported elevated heparanase levels and activity with progression of coronary atherosclerotic lesions from minimal lesions to thin cap fibroatheromas demonstrating a role of heparanase in coronary artery disease. Similarly, Osterholm et al. [43] investigated the expression of the heparanase gene and protein in a biobank of human carotid endarterectomy samples obtained from patients undergoing surgery for symptomatic and asymptomatic carotid disease. Briefly, microarray analysis of RNA extracted from 127 human carotid plaques showed a 6.6-fold increase in levels of heparanase mRNA in comparison to control tissue from nonatherosclerotic iliac arteries. In addition, heparanase mRNA expression correlated with increased serum creatinine levels and a reduced glomerular filtration rate (GFR), in accordance with studies on the causal involvement of heparanase in the development of diabetic nephropathy [44]. In their study, Blich et al. provided evidence for the clinical significance of heparanase in atherosclerosis [45]. The authors examined heparanase expression in specimens of stable and vulnerable atherosclerotic plaques compared with control arteries and revealed weak staining of heparanase in the media of control and stable plaque lesions, likely decorating smooth muscle cells compared to strikingly intense staining of heparanase in the intima of vulnerable plaque lesions.

Several studies demonstrated important proinflammatory functions of heparanase, mediating inflammatory and sepsis processes, mainly in lung and pancreas infections and inflammations, and also demonstrated a possible role of heparanase in atherosclerosis processes [46, 47].

Apolipoprotein E deficient mice (ApoE^−/−^ or E_0_ mice) are widely used as atherosclerotic animal model, since they develop severe hypercholesterolemia on a regular chow diet [48].

PG545 is a heparan sulphate mimetic that inhibits heparanase, the only endoglycosidase which cleaves heparan sulphate chains in the ECM [49, 50].

The extent of heparanase involvement in atherosclerosis progression has not been studied yet, and it is not clear whether inhibiting heparanase activity would result in reduction of oxidative stress and the resulting attenuation of atherosclerosis progression or not. Likewise, effects of heparanase inhibition on blood pressure and on serum glucose levels have not been studied yet.

## 2. Objectives

The aim of our study was to investigate the effect of inhibiting heparanase activity on oxidative stress, blood pressure, and serum glucose levels in E_0_ knockout transgenic mice.

## 3. Materials and Methods

### 3.1. Animal Studies

E_0_ mice were provided by courtesy of Professor Jan Breslow, Rockefeller University, NY, and they were bred and housed in pathogen-free conditions at the Animal Care Facility of the Rappaport Faculty of Medicine. 24 E_0_ male mice, 6-7 weeks of age, were divided into two matching groups, 12 mice in each. One group served as sham, control group, and were treated with weekly intraperitoneal normal saline injections for seven weeks. The second group of mice received heparanase inhibitor, PG545, administered on weekly basis using intraperitoneal injections for seven weeks, at the dosage of 0.4 mg/mouse [33]. Blood pressure was measured at the end of the last week of treatment, and the next day mice were sacrificed. Blood was obtained from all animals by direct puncture of hearts and aortas, where the samples were then centrifuged and serum was used for biochemical measurements.

### 3.2. Mice Blood Pressure Measurements

Computerized blood pressure measurements were performed (15–45 measurements per mouse) using the “CODA System,” a tail-cuff method, based on volume-pressure recording (VPR). The CODA System (Kent Scientific, Torrington, CT 06790, United States) is a computerized, noninvasive tail-cuff blood pressure measurement system that can perform single or multiple blood pressure measurements without operator intervention. The results are displayed as data plots and summary of digital values (systolic, diastolic, and mean blood pressure, in addition to heart rate, blood flow, and volume) [51].

### 3.3. Serum Analysis

#### 3.3.1. Biochemical Parameters

Serum creatinine, glucose, blood urea nitrogen (BUN), alanine aminotransferase (ALT), aspartate aminotransferase (AST), gamma glutamyl transferase (GGT), alkaline phosphatase (ALP), cholesterol, triglycerides, and HDL-cholesterol were determined by commercial kits (Siemens, Germany) using an autoanalyzer dedicated instrument (Dimension RXL, Siemens, Germany).

#### 3.3.2. Oxidative Stress

Serum analysis of lipid peroxidation: blood was collected into Eppendorf tubes by direct puncture of the aortas and the hearts after animals were sacrificed. Serum lipid peroxide content was determined using the thiobarbituric reactive substance (TBARS) assay [52, 53].

### 3.4. Statistical Analysis

Student's *t*-test and one-way analysis of variance (ANOVA) for repeated measures, followed by the Dunnett test, were used for comparison of treatment values with baseline in each group or with corresponding values in control group. All data analyses were conducted using GraphPad Prism (GraphPad Software, Inc., CA 92037, USA). All graphs were created using OriginLab, Data Analysis and Graphing Software (OriginLab Corporation, MA 01060, USA). A value of *p* < 0.05 was considered statistically significant. Data are presented as mean ± SEM.

## 4. Results

### 4.1. Blood Pressure

**Figure 1:**
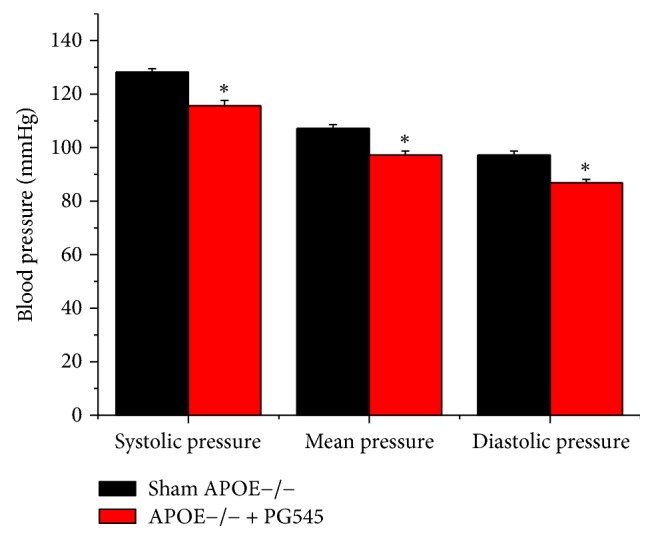
Effect of PG545 on mice blood pressure in APOE^−/−^ mice. Systolic, diastolic, and mean arterial pressure (mmHg) were evaluated following six weeks of treatment using the “CODA System” (*n* = 12 mice in each group), ^*∗*^*p* < 0.05.

Blood pressure was measured at the end of the treatment period. Administration of the heparanase inhibitor PG545 resulted in significant reduction in blood pressure. The systolic blood pressure was reduced significantly by 13 mmHg, from 128 ± 16 mmHg in the control group (*n* = 12) compared to 116 ± 23 mmHg in the study group (*n* = 12), the diastolic blood pressure by 11 mmHg, 97 ± 13 mmHg in the control group compared to 87 ± 12 in the study group, and the mean blood pressure by 10 mmHg, 107 ± 13 mmHg in the control group compared to 97 ± 20 mmHg in the treatment group, *p* < 0.001 compared to the control group in the three mentioned parameters (Figure 1).

### 4.2. Serum Analysis

#### 4.2.1. Blood Glucose

**Figure 2:**
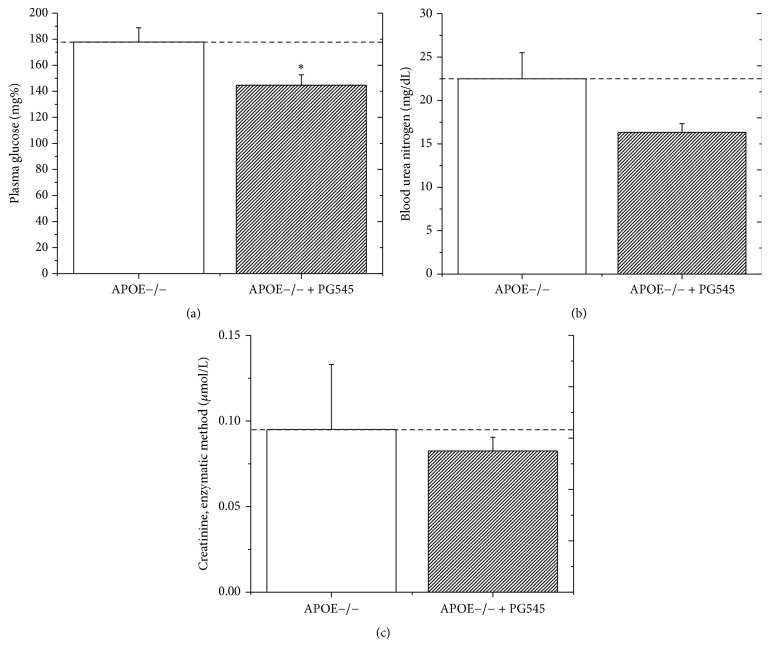
Effect of PG545 on selected biochemical parameters in APOE^−/−^ mice. Effect of PG545 on plasma glucose concentration (mg/dl) (a), blood urea nitrogen (mg/dl) (b), and plasma creatinine (*μ*mol/L) (c) in APOE−/− mice (*n* = 12 mice in each group), ^*∗*^*p* < 0.05.

At the end of the study, average blood glucose was 177 ± 10 mg/dl in the control group compared to 144 ± 11 mg/dl in the treatment group, a significant reduction of 19% in the treatment group compared to the control group (*p* < 0.05, Figure 2(a)).

#### 4.2.2. Kidney Function Tests

Administration of PG545 to mice caused a modest reduction in blood urea nitrogen and creatinine values, by 27% and 13% in the treatment group compared to the control group, respectively (*p* = 0.07, Figures 2(b) and 2(c)).

#### 4.2.3. Lipid Profile

Administration of PG545 caused a nonsignificant elevation of the total cholesterol level (452 ± 38 mg/dl in the treatment group compared to 363 ± 28 mg/dl in the control group, *p* = 0.082) and of the triglycerides levels (307 ± 48 mg/dl in the treatment group versus 207 ± 16 mg/dl in the control group, *p* = 0.057) and caused a significant decrease of the HDL-cholesterol levels: 96 ± 3 mg/dl in the treatment group compared to 127 ± 5 mg/dl in the control group, *p* < 0.001 (data not shown).

#### 4.2.4. Liver Function Tests

The liver enzymes ALT, AST, GGT, and ALP were measured for safety concerns regarding the use of the heparanase inhibitor.

**Figure 3:**
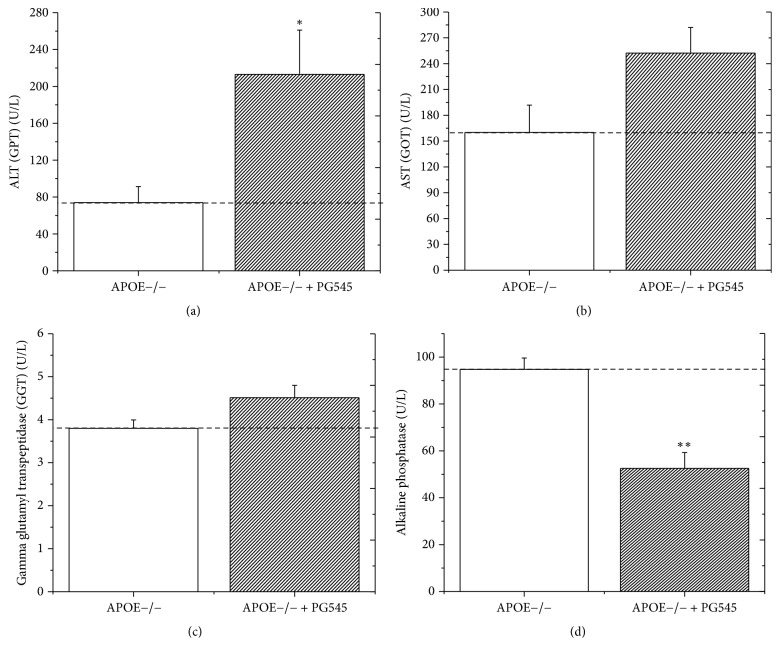
Effect of PG545 on liver enzymes profile in APOE^−/−^ mice. Effect of PG545 on ALT (U/L) (a), AST (U/L) (b), GGT (U/L) (c), and alkaline phosphate (U/L) (d) in APOE^/−^ mice (*n* = 12 mice in each group), ^*∗*^*p* < 0.05, ^*∗∗*^*p* < 0.01.

Administration of PG545 resulted in a significant elevation of the ALT (from 74 ± 17 U/L in the control group to 213 ± 47 U/L in the treatment group, an elevation of 187%, *p* < 0.05). Likewise, the AST was elevated in the treatment group, from 159 ± 32 U/L to 347 ± 97 U/L, but was statistically nonsignificant, *p* = 0.08. Interestingly, the cholestatic enzymes showed an inconsistent behavior and the GGT showed a minimal elevation (4.5 ± 0.3 U/L in the treatment group compared to 3.8 ± 0.2 U/L in the control group), while the ALP was significantly reduced in the treatment group compared to the control group, 52 ± 7 U/L compared to 94 ± 5 U/L, respectively, a 45% reduction (*p* < 0.01, Figure 3).

#### 4.2.5. Lipid Peroxidation

**Figure 4:**
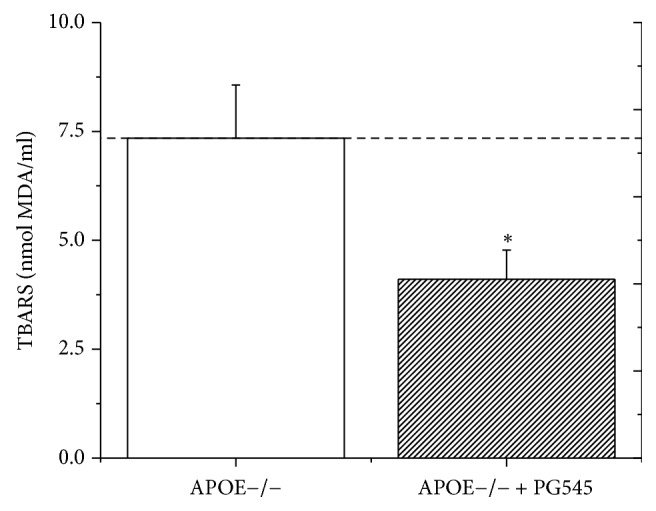
Serum oxidative stress measured by serum TBARS levels (nmol MDA/ml). Serum oxidative stress was evaluated using lipid peroxidation assay (TBARS) in APOE^−/−^ mice following PG545 administration (*n* = 12 mice in each group), ^*∗*^*p* < 0.05.

Decreased OS was observed in the treatment group compared to control group; PG545 reduced the thiobarbituric reactive substances (TBARS) serum levels by 44%, from 7.34 ± 1.2 nmol MDA/ml in the control group to 4.1 ± 0.6 nmol MDA/ml in the treatment group (*p* < 0.01, Figure 4).

#### 4.2.6. Body Weight

**Figure 5:**
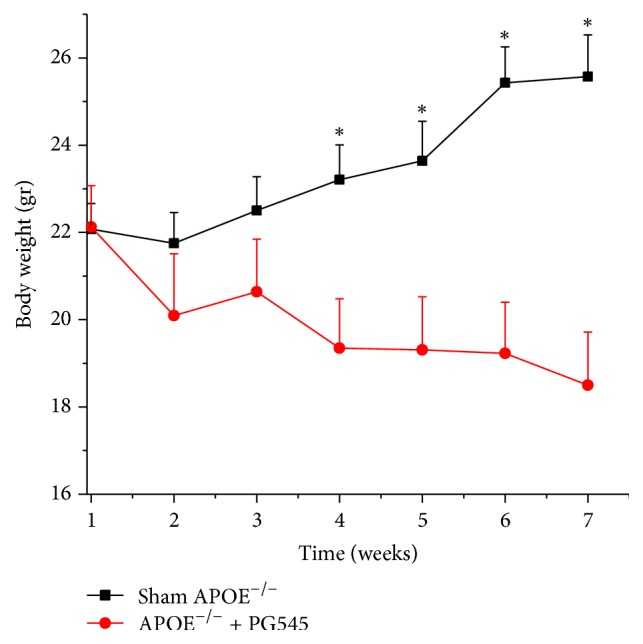
Effect of PG545 on mice body weight (gr) along the study. Body weight assessment through the seven weeks of study following PG545 administration (*n* = 12 mice in each group), ^*∗*^*p* < 0.05.

The average body weights of the animals at the beginning of the study were comparable in both groups, 22 ± 0.6 grams per animal in the control group and 22.1 ± 0.9 grams per animal in the treatment group. A prominent and consistent finding was the effect of heparanase inhibition on the mice body weight. While mice in the control group exhibited an average increase of 16% in their body weight compared to baseline, from 22 grams to 25.6 grams per animal on average, mice in the treatment group experienced a significant decrement in their body weight of 16%, from 22.1 grams per animal at the beginning of the study to 18.5 grams on average at the end of the study (*p* < 0.01). The weight loss effect was consistent along the study period starting on the measurements at week 4 (Figure 5).

## 5. Discussion

### 5.1. Oxidative Stress

 Oxidative stress is increased in patients with hypertension and diabetes mellitus and is even higher when blood pressure and diabetes mellitus are not well controlled or other disease-related complications present. Better control of diabetes mellitus and hypertension is thus accompanied by lower oxidative stress and lower rate of the expected complications [7–9]. In our study, heparanase inhibition in E_0_ mice caused significant lowering of blood pressure (both systolic and diastolic) and of serum glucose levels, which independently are expected to result in an improved prognosis. There are no reports in the literature on the effect of heparanase inhibition on blood pressure values. Interestingly, Shafat et al. [40] described markedly elevated urine and plasma levels of heparanase in diabetic patients compared to nondiabetic healthy subjects and reported an association between urine heparanase levels and blood glucose levels in the above-mentioned study. The authors concluded that higher glucose levels mediate heparanase upregulation and secretion into urine and blood and that heparanase could imply direct involvement in diabetic complications. In fact, diabetic heparanase null mice failed to develop diabetic nephropathy in response to streptozotocin [44]. It could be postulated that the oxidative stress lowering effect of the heparanase inhibitor could be secondary to lowering of blood pressure and of serum glucose levels. However, taking into consideration the fact that controlling diabetes is expected to be accompanied with slight weight gain, as insulin is known to be an anabolic hormone, in our study we postulate that the animals that were treated with PG545 exhibited a catabolic state, expressed by signs of possible lipolysis and significant weight loss. Additional studies are needed to investigate the effect of heparanase inhibition (possibly in lower doses) on the oxidative stress status regardless of the hypotensive and hypoglycemic effects of the inhibitor.

The proatherogenic properties of HSPG and of heparanase have been well studied. In their review, Wight and Merrilees reported that versican, one of the principal proteoglycans in the ECM of normal human blood vessels, accumulates in atherosclerotic lesions. The authors report that versican levels are dramatically increased in all forms of vascular disease and that it plays an important role in regulating many of the events that lead to the formation of atherosclerotic and restenotic lesions. The authors noted that versican tends to accumulate in human vessels susceptible to atherosclerosis, such as the coronary arteries and the saphenous veins, in comparison to atherosclerosis-resistant vessels, such as the internal mammary and the radial arteries [54]. Since heparanase is involved in ECM remodelling, we assume that this enzyme affects versican metabolism; therefore PG545 may exert its beneficial effect via inhibition of versican accumulation in blood vessels walls.

In another study, Vikramadithyan et al. [55] studied the role of perlecan, a dominant HSPG found in the subendothelial matrix of the vessel wall, in atherosclerosis development and progression in E_0_ mice. They reported higher levels of the HSPG in atherosclerotic lesions and concluded that the HSPG might mediate multiple processes that contribute to accelerated atherosclerosis. Baker et al. [42] demonstrated a critical role of heparanase in both the formation and the progression of vulnerable atherosclerotic plaques in diabetic-hyperlipidemic swine. In the latter study, immunohistochemical analysis revealed a pattern of heparanase accumulation along with lipid deposition and inflammatory cell distribution, suggesting that the major source of heparanase was the inflammatory cells. Notably, Blich et al. reported that heparanase immunostaining was markedly increased in vulnerable plaque specimens compared with stable plaque or control artery, also reflected by a ninefold increase of heparanase levels in the plasma of patients with acute myocardial infarction versus healthy subjects [45]. So far, there are no studies that evaluated the effects of heparanase inhibition on the oxidative stress.

### 5.2. Kidney Function

 Kidney function was assessed for safety concerns. Creatinine values were slightly lower in the study group compared to the control group, even though the difference was statistically nonsignificant. In a previous study, we reported a major role of heparanase in acute ischemia/reperfusion acute kidney injury in a mouse model, and treating animals with the heparanase inhibitors PG545 (in animals) and SST0001 (in cell culture) was associated with significantly reduced levels of markers of acute kidney injury [33]. We conclude that heparanase inhibition possibly provides a protective role against kidney function deterioration. In diabetic subjects, kidney injury is more frequent than in nondiabetic subjects, so the use of PG545 is expected to have a still more favorable effect in this patient population.

### 5.3. Serum Lipid Profile

The use of PG545 was associated with deleterious changes in the serum lipid profile compared to placebo. This finding is a matter of argument, as we performed lipid profile testing while animals were not fasting, a fact that may at least partially lead to the high triglycerides and total cholesterol levels, together with lower HDL levels. For accurate lipid profile testing, animals should be kept under overnight fasting status, but in our study this was not done and animals were under regular diet with free access to food and water until shortly before they were sacrificed. It would have been more appropriate to deprive animals from food overnight. Further studies should be held with mice kept in complete overnight fasting state in order to enable an accurate assessment of the effects of heparanase inhibition on the lipid profile.

### 5.4. Heparanase Inhibition and Atherosclerosis

In our study we did not evaluate the direct effect of heparanase inhibition on attenuation of atherosclerosis. It is expected that PG545 would cause attenuation of the atherosclerosis process, given that Blich et al. [45] described accelerated risk of acute MI along with higher heparanase levels. It was also reported that heparanase induces activation of macrophages and exerts a strong angiogenic response. Moreover, the authors also noted that heparanase was associated with accelerated proliferation of smooth muscle cells, implying that the enzyme affects all major cellular components (i.e., endothelial cells, vascular smooth muscle cells, and macrophages) of the atherosclerotic lesion [45]. Overexpression of the heparanase gene revealed the importance of HSPGs for the uptake of triglyceride rich lipoproteins (TRPs) and their protective effect on fatty streak formation and potentially atherosclerosis initiation [23]. Whether inhibition of heparanase causes attenuation of atherosclerosis progression or not has not been studied so far.

### 5.5. Safety Concerns

#### 5.5.1. Weight Loss

The use of PG545 was associated with significant weight loss in comparison to placebo. In their study, Karlsson-Lindahl et al. have described the role of heparanase in food intake behavior and regulation of energy balance in mice, presuming heparanase activity via the central melanocortin system [56]. In transgenic mice used in their study, overexpression of heparanase was associated with reduced body fat compared to wild type mice, but with no significant differences in the lean body mass or body weight. Inhibition of heparanase is therefore expected to result in weight gain, while in our study there was significant weight loss in all the animals that received the heparanase inhibitor. We did not assess the direct effect of the heparanase inhibitor on food intake.

#### 5.5.2. Hepatotoxicity

Only the ALT was significantly elevated, whereas AST and GGT were nonsignificantly elevated and, controversially, the ALP was significantly reduced. In literature search, no previous studies on heparanase inhibition and liver function tests were found. This hepatotoxic effect could be dose-related. Lower doses of the inhibitor should be tested in the future, as hepatotoxicity, as well as the deleterious effect on body weight, could be dose-related.

In summary, despite the encouraging results, one should not ignore the toxic potential of PG545, as was evident by its deleterious impact on the liver enzymes, lipid profile, and body weight of the animals. However, it should be emphasized that the PG545 toxicity could be attributed to the applied dose in the current study. Additional future studies should utilize either lower doses of the inhibitor, or using other heparanase inhibitors (such as SST0001), which unfortunately are not available commercially.

## 6. Conclusions

Our study demonstrated favorable impact of the heparanase inhibitor PG545 on blood pressure, serum glucose levels, and oxidative stress in apolipoprotein E deficient mice, and as a result one can anticipate a favorable effect towards attenuating atherosclerosis progression by the inhibitor. The possible antiatherosclerotic effect could be related to the better control of factors contributing to accelerated atherosclerosis, namely, high blood pressure, serum glucose levels, and oxidative stress load.

Safety concerns include prominent weight loss rather than the expected normal weight gain and hepatotoxicity expressed by a significant elevation of ALT but not the other hepatic enzymes. Of note is the significantly decreased alkaline phosphatase levels, and possibly altered lipid profile, namely, notable but nonsignificant elevation of TC and TG levels, together with a significant lowering of HDL-cholesterol levels. The latter effect is questionable, as blood was drawn while animals were not kept in the essential overnight fasting condition.

## References

[1] Halliwell B. (1994). Free radicals, antioxidants, and human disease: curiosity, cause, or consequence?. *The Lancet*.

[2] Kaplan M., Aviram M. (1999). Oxidized low density lipoprotein: Atherogenic and proinflammatory characteristics during macrophage foam cell formation. An inhibitory role for nutritional antioxidants and serum paraoxonase. *Clinical Chemistry and Laboratory Medicine*.

[3] Dragsted L. O. (2003). Antioxidant actions of polyphenols in humans. *International Journal for Vitamin and Nutrition Research*.

[4] Aviram M., Kaplan M., Rosenblat M., Fuhrman B. (2005). Dietary antioxidants and paraoxonases against LDL oxidation and atherosclerosis development. *Handbook of Experimental Pharmacology*.

[5] Nigdikar S. V., Williams N. R., Griffin B. A., Howard A. N. (1998). Consumption of red wine polyphenols reduces the susceptibility of low- density lipoproteins to oxidation in vivo. *American Journal of Clinical Nutrition*.

[6] Aviram M., Fuhrman B. (2002). Wine flavonoids protect against LDL oxidation and atherosclerosis. *Annals of the New York Academy of Sciences*.

[7] Aviram M., Dornfeld L., Rosenblat M. (2000). Pomegranate juice consumption reduces oxidative stress, atherogenic modifications to LDL, and platelet aggregation: studies in humans and in atherosclerotic apolipoprotein E-deficient mice. *American Journal of Clinical Nutrition*.

[8] Kaplan M., Hayek T., Raz A. (2001). Pomegranate juice supplementation to atherosclerotic mice reduces macrophage lipid peroxidation, cellular cholesterol accumulation and development of atherosclerosis. *Journal of Nutrition*.

[9] Aviram M., Rosenblat M., Gaitini D. (2008). Erratum in: Pomegranate juice consumption for 3 years by patients with carotid artery stenosis reduces common carotid intima-media thickness, blood pressure and LDL oxidation. *Clinical Nutrition*.

[10] Hamoud S., Hayek T., Volkova N. (2014). Pomegranate extract (POMx) decreases the atherogenicity of serum and of human monocyte-derived macrophages (HMDM) in simvastatin-treated hypercholesterolemic patients: A double-blinded, placebo-controlled, randomized, prospective pilot study. *Atherosclerosis*.

[11] Vaya J., Belinky P. A., Aviram M. (1997). Antioxidant constituents from licorice roots: Isolation, structure elucidation and antioxidative capacity toward LDL oxidation. *Free Radical Biology & Medicine*.

[12] Belinky P. A., Aviram M., Fuhrman B., Rosenblat M., Vaya J. (1998). The antioxidative effects of the isoflavan glabridin on endogenous constituents of LDL during its oxidation. *Atherosclerosis*.

[13] Hamoud S., Kaplan M., Meilin E. (2013). Niacin administration significantly reduces oxidative stress in patients with hypercholesterolemia and low levels of high-density lipoprotein cholesterol. *The American Journal of the Medical Sciences*.

[14] Bernfield M., Götte M., Park P. W. (1999). Functions of cell surface heparan sulfate proteoglycans. *Annual Review of Biochemistry*.

[15] Ilan N., Elkin M., Vlodavsky I. (2006). Regulation, function and clinical significance of heparanase in cancer metastasis and angiogenesis. *The International Journal of Biochemistry & Cell Biology*.

[16] Sasisekharan R., Shriver Z., Venkataraman G., Narayanasami U. (2002). Roles of heparan-sulphate glycosaminoglycans in cancer. *Nature Reviews Cancer*.

[17] Vlodavsky I., Friedmann Y. (2001). Molecular properties and involvement of heparanase in cancer metastasis and angiogenesis. *The Journal of Clinical Investigation*.

[18] Parish C. R., Freeman C., Hulett M. D. (2001). Heparanase: A key enzyme involved in cell invasion. *Biochimica et Biophysica Acta (BBA) - Reviews on Cancer*.

[19] Barash U., Cohen-Kaplan V., Dowek I., Sanderson R. D., Ilan N., Vlodavsky I. (2010). Proteoglycans in health and disease: New concepts for heparanase function in tumor progression and metastasis. *FEBS Journal*.

[20] Sandwall E., O'Callaghan P., Zhang X., Lindahl U., Lannfelt L., Li J. (2010). Heparan sulfate mediates amyloid-beta internalization and cytotoxicity. *Glycobiology*.

[21] Zetser A., Bashenko Y., Edovitsky E., Levy-Adam F., Vlodavsky I., Ilan N. (2006). Heparanase induces vascular endothelial growth factor expression: Correlation with p38 phosphorylation levels and Src activation. *Cancer Research*.

[22] Vlodavsky I., Beckhove P., Lerner I. (2012). Significance of heparanase in cancer and inflammation. *Cancer Microenvironment*.

[23] Vlodavsky I., Blich M., Li J.-P., Sanderson R. D., Ilan N. (2013). Involvement of heparanase in atherosclerosis and other vessel wall pathologies. *Matrix Biology*.

[24] Lygizos M. I., Yang Y., Altmann C. J. (2013). Heparanase mediates renal dysfunction during early sepsis in mice. *Physiological Reports*.

[25] Goldberg R., Rubinstein A. M., Gil N. (2014). Role of heparanase-driven inflammatory cascade in pathogenesis of diabetic nephropathy. *Diabetes*.

[26] Sidaway P. (2014). Diabetic nephropathy: Heparanase mediates renal injury. *Nature Reviews Nephrology*.

[27] Tao Y. H., Wang Z., Zhou Y. R. (2014). Expression of heparanase in kidney of rats with respiratory syncytial virus nephropathy and its relationship with proteinurina. *Sichuan Da Xue Xue Bao Yi Xue Ban*.

[28] Assady S., Alter J., Axelman E. (2015). Nephroprotective effect of heparanase in experimental nephrotic syndrome. *PLoS ONE*.

[29] Szymczak M., Kuźniar J., Klinger M. (2010). The role of heparanase in diseases of the glomeruli. *Archivum Immunologiae et Therapia Experimentalis*.

[30] Van Den Hoven M. J., Rops A. L., Vlodavsky I., Levidiotis V., Berden J. H., Van Der Vlag J. (2007). Heparanase in glomerular diseases. *Kidney International*.

[31] Levidiotis V., Freeman C., Tikellis C., Cooper M. E., Power D. A. (2004). Heparanase is involved in the pathogenesis of proteinuria as a result of glomerulonephritis. *Journal of the American Society of Nephrology*.

[32] Levidiotis V., Kanellis J., Ierino F. L., Power D. A. (2001). Increased expression of heparanase in puromycin aminonucleoside nephrosis. *Kidney International*.

[33] Masola V., Zaza G., Gambaro G. (2016). Heparanase: a potential new factor involved in the renal epithelial mesenchymal transition (EMT) induced by ischemia/reperfusion (I/R) injury. *PLoS ONE*.

[34] Kramer A., Van Den Hoven M., Rops A. (2006). Induction of glomerular heparanase expression in rats with adriamycin nephropathy is regulated by reactive oxygen species and the renin-angiotensin system. *Journal of the American Society of Nephrology*.

[35] Van Den Hoven M. J., Waanders F., Rops A. L. (2009). Regulation of glomerular heparanase expression by aldosterone, angiotensin II and reactive oxygen species. *Nephrology Dialysis Transplantation *.

[36] Levidiotis V., Freeman C., Tikellis C., Cooper M. E., Power D. A. (2005). Heparanase inhibition reduces proteinuria in a model of accelerated anti-glomerular basement membrane antibody disease. *Nephrology*.

[37] Van Den Hoven M. J., Rops A. L., Bakker M. A. (2006). Increased expression of heparanase in overt diabetic nephropathy. *Kidney International*.

[38] Rops A. L. W. M. M., Van Den Hoven M. J., Veldman B. A. (2012). Urinary heparanase activity in patients with Type 1 and Type 2 diabetes. *Nephrology Dialysis Transplantation *.

[39] Shafat I., Agbaria A., Boaz M. (2012). Elevated urine heparanase levels are associated with proteinuria and decreased renal allograft function. *PLoS ONE*.

[40] Shafat I., Ilan N., Zoabi S., Vlodavsky I., Nakhoul F. (2011). Heparanase levels are elevated in the urine and plasma of type 2 diabetes patients and associate with blood glucose levels. *PLoS ONE*.

[41] Celie J. W. A. M., Katta K. K., Adepu S. (2012). Tubular epithelial syndecan-1 maintains renal function in murine ischemia/reperfusion and human transplantation. *Kidney International*.

[42] Baker A. B., Chatzizisis Y. S., Beigel R. (2010). Regulation of heparanase expression in coronary artery disease in diabetic, hyperlipidemic swine. *Atherosclerosis*.

[43] Österholm C., Folkersen L., Lengquist M. (2013). Increased expression of heparanase in symptomatic carotid atherosclerosis. *Atherosclerosis*.

[44] Gil N., Goldberg R., Neuman T. (2012). Heparanase is essential for the development of diabetic nephropathy in mice. *Diabetes*.

[45] Blich M., Golan A., Arvatz G. (2013). Macrophage activation by heparanase is mediated by TLR-2 and TLR-4 and associates with plaque progression. *Arteriosclerosis, Thrombosis, and Vascular Biology*.

[46] Li P., Burdmann E. A., Mehta R. L. (2013). Acute kidney injury: global health alert world kidney day steering committee. *Journal of Nephropathology*.

[47] Kaushal G. P., Shah S. V. (2014). Challenges and advances in the treatment of AKI. *Journal of the American Society of Nephrology*.

[48] Meir K. S., Leitersdorf E. (2004). Atherosclerosis in the apolipoprotein E-deficient mouse: a decade of progress. *Arteriosclerosis, Thrombosis, and Vascular Biology*.

[49] Dredge K., Hammond E., Handley P. (2011). PG545, a dual heparanase and angiogenesis inhibitor, induces potent anti-tumour and anti-metastatic efficacy in preclinical models. *British Journal of Cancer*.

[50] Ferro V., Liu L., Johnstone K. D. (2012). Discovery of PG545: A highly potent and simultaneous inhibitor of angiogenesis, tumor growth, and metastasis. *Journal of Medicinal Chemistry*.

[51] De Frutos S., Duling L., Alò D. (2008). NFATc3 is required for intermittent hypoxia-induced hypertension. *American Journal of Physiology-Heart and Circulatory Physiology*.

[52] Aviram M., Vaya J. (2001). Markers for low-density lipoprotein oxidation. *Methods in Enzymology*.

[53] Buege J. A., Aust S. D. (1978). Microsomal lipid peroxidation. *Methods in Enzymology*.

[54] Wight T. N., Merrilees M. J. (2004). Proteoglycans in atherosclerosis and restenosis: Key roles for versican. *Circulation Research*.

[55] Vikramadithyan R. K., Kako Y., Chen G. (2004). Atherosclerosis in perlecan heterozygous mice. *Journal of Lipid Research*.

[56] Karlsson-Lindahl L., Schmidt L., Haage D. (2012). Heparanase affects food intake and regulates energy balance in mice. *PLoS ONE*.

